# Cathelicidin-Derived Antimicrobial Peptides Inhibit Zika Virus Through Direct Inactivation and Interferon Pathway

**DOI:** 10.3389/fimmu.2018.00722

**Published:** 2018-04-12

**Authors:** Miao He, Hainan Zhang, Yuju Li, Guangshun Wang, Beisha Tang, Jeffrey Zhao, Yunlong Huang, Jialin Zheng

**Affiliations:** ^1^Department of Neurology, Xiangya Hospital, Central South University, Changsha, China; ^2^Department of Pharmacology and Experimental Neuroscience, University of Nebraska Medical Center, Omaha, NE, United States; ^3^Department of Neurology, The Second Xiangya Hospital, Central South University, Changsha, China; ^4^Center for Translational Neurodegeneration and Regenerative Therapy, Shanghai Tenth People’s Hospital Affiliated to Tongji University School of Medicine, Shanghai, China; ^5^Department of Pathology and Microbiology, University of Nebraska Medical Center, Omaha, NE, United States; ^6^National Clinical Research Center for Geriatric Diseases, Changsha, China; ^7^Key Laboratory of Hunan Province in Neurodegenerative Disorders, Central South University, Changsha, China

**Keywords:** antimicrobial peptides, Zika virus, innate immunity, cathelicidins, plaque-forming assays

## Abstract

Zika virus (ZIKV) is a neurotrophic flavivirus that is able to infect pregnant women and cause fetal brain abnormalities. Although there is a significant effort in identifying anti-ZIKV strategies, currently no vaccines or specific therapies are available to treat ZIKV infection. Antimicrobial peptides, which are potent host defense molecules in nearly all forms of life, have been found to be effective against several types of viruses such as HIV-1 and influenza A. However, they have not been tested in ZIKV infection. To determine whether antimicrobial peptides have anti-ZIKV effects, we used nine peptides mostly derived from human and bovine cathelicidins. Two peptides, GF-17 and BMAP-18, were found to have strong anti-ZIKV activities and little toxicity at 10 µM in an African green monkey kidney cell line. We further tested GF-17 and BMAP-18 in human fetal astrocytes, a known susceptible cell type for ZIKV, and found that GF-17 and BMAP-18 effectively inhibited ZIKV regardless of whether peptides were added before or after ZIKV infection. Interestingly, inhibition of type-I interferon signaling resulted in higher levels of ZIKV infection as measured by viral RNA production and partially reversed GF-17-mediated viral inhibition. More importantly, pretreatment with GF-17 and BMAP-18 did not affect viral attachment but reduced viral RNA early in the infection course. Direct incubation with GF-17 for 1 to 4 h specifically reduced the number of infectious Zika virions in the inoculum. In conclusion, these findings suggest that cathelicidin-derived antimicrobial peptides inhibit ZIKV through direct inactivation of the virus and *via* the interferon pathway. Strategies that harness antimicrobial peptides might be useful in halting ZIKV infection.

## Introduction

Zika virus (ZIKV) is an enveloped neurotrophic flavivirus mainly transmitted by *Aedes* mosquitoes (1). Since early 2015, the rapid spread of ZIKV in South America is known to cause increased incidence of intrauterine infection that is associated with fetal microcephaly and other congenital malformations (2–7). There is also strong evidence to link ZIKV with Guillain–Barré syndrome (GBS), which is an autoimmune disorder that causes demyelination in peripheral nerves (8–11). In addition, meningitis and meningoencephalitis have also been described in adults with history of ZIKV infection (12). Although there are much fewer cases of vector-borne ZIKV infection in the epidemic area since February of 2017, potential for new epidemics beyond currently impacted countries remains high. The epidemics of ZIKV have brought increased urgency to the development of safe and effective anti-ZIKV drugs. However, currently no vaccines or specific therapies are available for the prevention or treatment of ZIKV infection.

Antimicrobial peptides (AMPs) are a group of relatively short (usually less than 50 amino acids), cationic (net charge of + 1 to + 7), and amphipathic peptides that constitute part of innate immune molecules in nearly all forms of life (13–15). These peptides may be expressed constitutively or induced in response to infectious/inflammatory stimuli, and play an important role in eliminating invading pathogenic microorganisms, including bacteria, fungi, and viruses. Several AMPs also have selective inhibitory effects on tumor growth (16). Although most AMPs have limited sequence and structure homologies, they are grouped into families based on their sequence and structural similarities (15). In mammals, cathelicidins and defensins are two major families of AMPs. In humans, there are also other AMP families, including lysozyme, dermcidin, and histatin (17).

Cathelicidins are generally located at the C-termini of a 15–18 kDa precursors that share a highly conserved domain called cathelin (acronym for cathepsin L inhibitor) (18). In humans, LL-37, a 37- amino-acid cationic peptide starting with a pair of leucines, is a widely studied peptide derived from the only cathelicidin gene (19). LL-37 is well documented in host defense against a variety of microbial infections (20–24). However, the effect of LL-37 has not been tested in ZIKV infection. In the current study, we hypothesize that cathelicidin-derived peptides are effective in inhibiting ZIKV infection. We examined nine different peptides mostly derived from human and bovine cathelicidins and found that several of them have anti-ZIKV activities *in vitro*. We elucidated that the mechanisms of action of these cathe-licidin-derived peptides are through direct inactivation and/or the activation of type-I interferon (IFN) signaling.

## Materials and Methods

### Ethics Statement

All experiments for human fetal astrocyte generation were performed with the approval of the Scientific Research Oversight Committee at the University of Nebraska Medical Center (UNMC). Human fetal brain tissues were obtained from elective aborted specimens (gestational age 12 weeks to 16 weeks) following completion of the abortion procedure through collaborative works with the Birth Defects Research laboratory at University of Washington. The protocol is in compliance with all relevant state and federal regulations and is approved by the University of Washington Institutional Review Board (IRB, Protocol no. 96-1826-A07) and UNMC IRB (Protocol no. 123-02-FB). Written informed consent was obtained with all subjects using an IRB-approved consent form at the University of Washington. All consenting subjects were donors of fetal tissue that were 19 years of age or older with clear comprehension. The UNMC investigators do not have access to signed consent forms.

### Antibodies and Reagents

Anti-human IFN-α polyclonal neutralizing antibody was purchased from R&D Systems and used at 200 neutralizing units/mL (R&D, Minneapolis, MN, USA, 31130-1). Rabbit IgG control (R&D, AB-105-C) served as the control for IFN-α neutralizing antibody. Fludarabine (Sigma, F2773) was purchased from Sigma-Aldrich (Sigma, St. Louis, MO, USA). Dimethyl sulfoxide (DMSO, Sigma, D4540) served as the solvent control for fludarabine.

### Cells and Zika Viruses

Human fetal astrocytes were derived from a single-cell isolation process of fetal brain tissues as previously described (25, 26). Briefly, dissociated brain tissue was incubated with 0.25% trypsin for 30 min, followed by neutralization with 10% fetal bovine serum (FBS), and further dissociated by trituration. The single-cell suspension was cultured as adherent cells in DMEM/F12 (Thermo Fisher Scientific, Waltham, MA, USA), supplemented with 10% FBS, penicillin (50 units/mL), and streptomycin (50 µg/mL) (Thermo Fisher Scientific). This process yields a culture of >95% glial fibrillary acidic protein (GFAP, Dako Corp., Carpinteria, CA, USA) positive astrocytes in immunocytochemical staining. Vero cells (ATCC, CCL-81), an African green monkey kidney cell line, were maintained in Dulbecco’s Modified Eagle Medium (DMEM) with 5% FBS. Experiments with ZIKV were performed exclusively inside a BioSafety Level 2 (laboratory). All procedures utilized in this study were approved by the Institutional Biosafety Committee (IBC 16-05-013BL2) and followed biosafety level II practices as shown in National Institutes of health (NIH) Guideline Appendix G-II-B. ZIKV strain MR766 (Uganda, 1947) was obtained from ZeptoMetrix Corp., Buffalo, NY, USA, and propagated in Vero cells. ZIKV infection of the Vero cells and fetal astrocytes was previously characterized (27) and all infections in the current studies were at the MOI of 0.5.

### Antimicrobial Peptides

**Figure 1:**
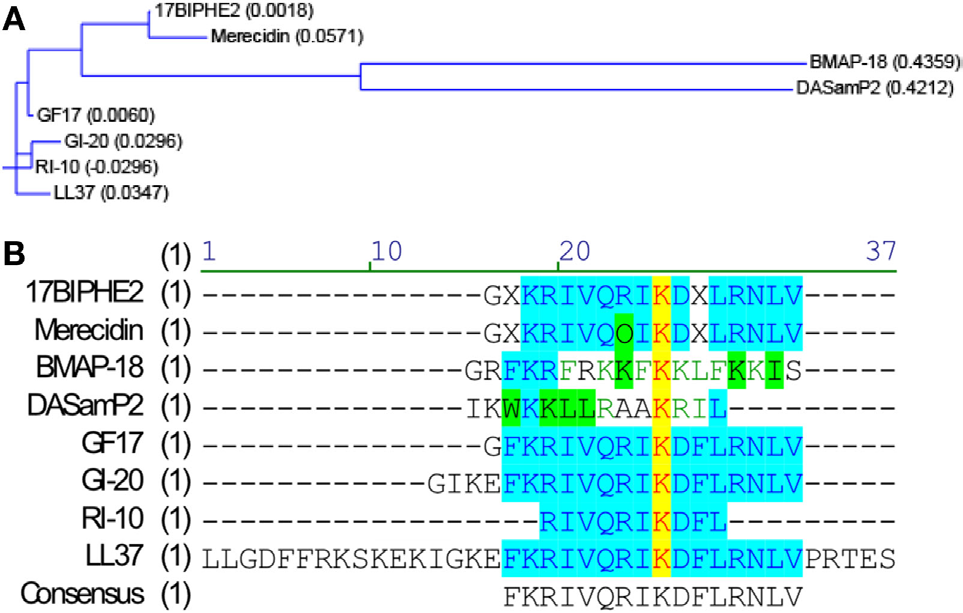
Sequence relationships of the nine peptides. Sequence relationships of the nine antimicrobial peptides (AMPs). Amino-acid sequences of the AMPs were compared with the Vector NTI software suite (Thermo Fisher Scientific). **(A)** Working relationships between the nine AMPs derived from human and bovine cathelicidins are shown in the dendrogram based on the amino-acid sequence homology. The length of each tree branch is proportional to the number of amino-acid differences between a particular sequence and its theoretical ancestral sequence (presented as parentheses for each sequence). **(B)** Amino-acid sequence alignment. Color codes indicate degree of similarity: yellow, font red: identical; cyan, font blue: conservative; green, font black: similar; no color, font green: weakly similar, no color, font black: non-similar.

To identify potent AMPs, we used two major methods: (1) structure-based design and (2) database screening. Through structural studies of human cathelicidin LL-37 and its fragments, we identified and designed a series of peptides. We also used a database approach to identify potent antibacterial and antiviral peptides (28–31). Nine AMPs used in this study include human cathelicidin LL-37, LL-37-derived peptides (GI-20, GI-20D-form, GF-17, 17BIPHE2, Merecidin, RI-10), bovine cathelicidin BMAP-27-derived BMAP-18 (32), and DASamP2 (30) (Figure 1). GI-20 corresponds to residues 13–32 of LL-37 with the positions of the first two residues IG swapped. A D-form of GI-20 was also synthesized using entirely D-amino acids (32). GF-17 corresponds to the major antimicrobial region (residues 17–32) of LL-37 (28). RI-10, which lacks antibacterial activity, shares part of the sequence of GF-17 (29). Peptide 17BIPHE2 is derived from GF-17 to gain stability to proteases such as chymotrypsin (33), while merecidin is a derivative of 17BIPHE2 (31). In the sequence of 17BIPHE2, X represents biphenylalanine and the letter O in merecidin stands for ornithine. Likewise, BMAP-18 corresponds to the N-terminal portion of bovine cathelicidin BMAP-27 with residue L17 replaced by I17 (34). DASamP2 is an AMP against methicillin-resistant *Staphylococcus aureus* (MRSA) and *Pseudomonas aeruginosa* identified by screening representative peptides selected from the AMP database (*http://aps.unmc.edu/AP*) (15).

### Cell Viability Assay

Cell viability was examined by the CellTiter 96 ^®^Aqueous One Solution Cell Proliferation Assay (Promega, Madison, WI, USA). Vero cells and astrocytes were seeded into 96-well tissue culture plates (Fisher Scientific) and treated with fresh growth medium containing different concentrations of each peptide for 2 or 4 days at 37°C. At the experimental end point, MTS [3-(4,5-dimethylthiazol-2-yl)-5-(3-carboxymethoxyphenyl)-2-(4-sulfophenyl)-2H-tetrazolium] was added to the cultures and incubated for 1 h. The absorbance at 490 nm was recorded through BioTek Gen5 data analysis software (Winooski), VT, optical density (OD) values were used as a representation of cell viability.

### ZIKV Plaque-Forming Assay

Vero cells were plated into 12-well plates at 5 (105 cells/well the day before infection). On the day of infection, the monolayers of Vero cells were inoculated with 100 µL of 10-fold serial dilutions of viral stocks and incubated at 37oC for 1 h. After viral inoculation, medium containing ZIKV particles was removed and 1 ml overlay containing 0.6% molecular biology grade agarose (Agarose Unlimited, Alachua, FL, USA) in Modified Eagle Medium (Gibco) with 2% FBS. Cells were maintained at 37°C in 5% CO_2_ for 4 days. On day 5, cells were fixed with 4% paraformaldehyde solution in PBS and stained with 1% crystal violet solution in 20% methanol in water. Viral plaques were photographed using a CanonScan 9950F scanner and each plaque was counted as a plaque-forming unit (PFU). Viral titer was calculated as PFU/[volume virus (mL) × (dilution factor)].

### Real-Time RT-PCR

Total mRNA was isolated with TRIzol Reagent (Thermo Fisher Scientific) and RNeasy Mini Kit (QIAGEN Inc., Valencia, CA, USA) following the manufacturer’s recommendations. Two real-time RT-PCR methods were used for the current studies. First, SYBR Green-based RT-PCR assay was used to determine intracellular ZIKV RNA in Vero cells. For this assay, reverse transcription was performed using Verso cDNA synthesis Kit (Thermo Fisher Scientific) and 1 µg of total RNA in a 15-µL final volume. The RT-PCR analyses were performed using 7.5-µL SYBR Green PCR Master Mix (Thermo Fisher Scientific) with 0.5-µL cDNA, 1.5-µL H_2_O, 5.5-µL oligonucleotide primer pairs at 10 µM. Primers used were ZIKV RNA: forward sequence 5-TGGGAGGTTTGAAGAGGCTG-3, reverse sequence 5-TCTCAACATGGCAGCAAGATCT-3; GAPDH: forward sequence 5-GGAGCGAGATCCCTCCAAAAT-3, reverse sequence 5-GGCTGT TGTCATACTTCTCAT GG-3. PCR program: 1, 50°C for 2 min; 2, 95°C for 2 min; 3, 95°C for 15 s; 4, specific annealing temperature for 15 s; 5, 72°C for 1 min. Steps 2–4 were repeated 40 times. All samples were amplified in triplicate for analysis. Relative ZIKV RNA levels were determined and standardized with a GAPDH internal control using comparative ΔΔCT method (35). Second, TaqMan-based RT-PCR assay was used to determine intracellular ZIKV RNA and other gene expressions in Figures 3–6 and extracellular ZIKV RNA in Figure 3. The TaqMan assay was performed in a StepOne^TM^ Real-Time PCR system (Thermo Fisher Scientific). Primers used for TaqMan real-time RT-PCR were all from the Thermo Fisher Scientific, which included ZIKV (forward sequence: 5-TTGGTCATGATACTGCTGATTGC-3, reverse sequence: 5- CCTTCCACAAAGTCCCTATTGC-3, and probe sequence: 5′-CGGCATACAGCATCAGGTGCATAGGAG-3), IFN-α2 (Hs00265051_s1), IFN-β1 (Hs01077958_s1), eukaryotic 18-s rRNA (Hs99999901_s1), β-actin (Hs99999903_m1), and GAPDH (Catalog number: 4310884E). Relative ZIKV mRNA levels were determined and standardized with a GAPDH or 18-s rRNA endogenous control using comparative ΔΔCT method (35). All reference genes, including GAPDH, β-actin, and 18-s rRNA, detected identical changes of genes (Figure S1 in Supplementary Material). Therefore, typically one was chosen as the endogenous control for gene expressions in the current studies. For ZIKV in supernatants, RNA was extracted from the supernatants through TRIzol™ LS Reagent (Thermo Fisher Scientific, 10296028) following the manufacturer’s manual. Since reference genes were not expressed in the supernatant RNA, an equal volume of extracted RNA was used in real-time RT-PCR. Quantitative genomic RNA from ZIKV (NR-1838DQ, ATCC) was used as a standard to calculate viral copies. All primers used in the study were tested for amplification efficiencies and the results were similar to each other.

### Viral Attachment Assay

Viral attachment assay was adapted from a previous publication (36) with modifications. Briefly, primary human fetal astrocytes were pre-incubated at 4°C for half an hour and then treated with AMPs along with ZIKV infection at the MOI of 0.5. After infection at 4°C for 2 h, cultures were washed with fresh medium for three times and RNA was isolated from whole cells. ZIKV RNA was determined through real-time RT-PCR. Data were normalized to 18-s rRNA and presented as fold change compared with the ZIKV group. One experimental group was subjected to trypsin digestion to remove any attached virions. This group served as a positive control for the viral attachment experiment.

### Direct Inactivation of ZIKV by AMPs

Zika virus viral stocks were diluted to 10^6^ PFU/mL with serum-free medium and peptides were added at the indicated final concentrations. The mixtures were incubated at 37°C for 1–4 h. At each time point, 200 µL of the mixtures was removed for virus yield determination. Ten-fold serial dilutions of the mixtures were prepared and number of infectious Zika virions in the inoculum was determined by the aforementioned plaque-forming assay on Vero cell monolayers.

### Statistical Analysis

Statistical analyses were performed using GraphPad Prism 7.00 and IBM SPSS Statistics Version 22. The data were presented as means ± SD unless specified otherwise. The EC_50_ values of cytotoxicity data were calculated using GraphPad Prism 7.00. Differences between groups were compared using the one-way ANOVA with Bonferroni post-test for multiple comparison. For the data that are not normally distributed due to varying levels of ZIKV inhibition, we applied the log transformation to meet normality of data distribution before statistical tests. *p* < 0.05 was considered as significant. All assays were performed at least three times in triplicate or quadruplicate.

## Results

### AMPs Inhibit ZIKV Infection in Vero Cells

**Figure 2:**
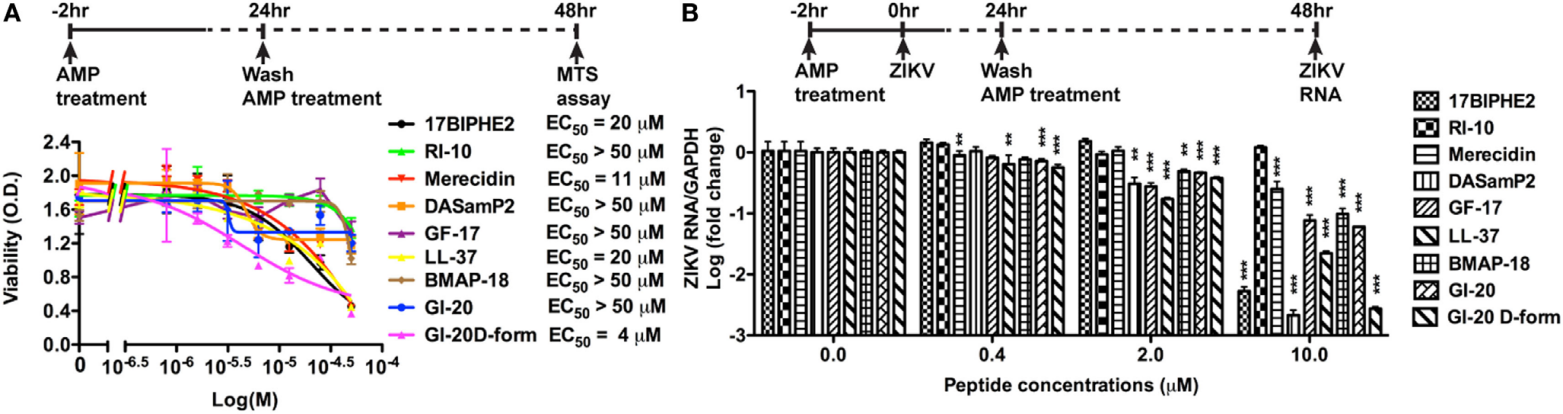
Antimicrobial peptides derived from cathelicidins inhibit Zika virus (ZIKV) infection in Vero cells. **(A)** Vero cells were treated with doses of peptides ranging from 1 to 50 µM. After 24 h, the cultures were washed and treated with same doses of peptides in fresh medium for another 24 h. Cell viability was determined by a colorimetric MTS assay. Results shown are the averages ±SD of experiments performed in triplicate. Data were analyzed by non-linear regression. The EC50 was assessed for each of the antimicrobial peptides. **(B)** Vero cells were treated with doses of peptides ranging from 0.4 to 10 µM for 2 h then infected with ZIKV at the MOI of 0.5. After 24 h, the cultures were washed and treated with same doses of peptides in fresh medium for another 24 h. ZIKA RNA was detected in total cellular RNA through real-time RT-PCR. Data were normalized to GAPDH and presented as fold change (in log scale) compared with ZIKV group. ** *p* < 0.01 and *** *p* < 0.001, as compared with the ZIKV group without the peptide treatment (ANOVA, *N* = 3).

To determine whether AMPs have anti-ZIKV effects *in vitro*, we selected nine peptides, including eight active AMPs and one non-antimicrobial control peptide––RI10. Most of these peptides are closely related to human cathelicidin LL-37. LL-37 is aligned with GI-20 (32), GF-17 (28), and RI-10 (29), which corresponds to residues 13–32, 17–32, and 19–28 of LL-37, respectively, while another two peptides, 17BIPHE2 and merecidin, are designed based on GF-17 to gain stability to proteases such as chymotrypsin (Figures 1A,B). In contrast, bovine cathelicidin-derived BMAP-18 and database-obtained DASamP2 are more distantly related to LL-37 compared with those aforementioned AMPs (30, 32). BMAP-18 and GF-17 share a similar amphipathic helical pattern, where four residues are identical and seven residues are semi-conserved (Figure 1). We first evaluated the cytotoxicity of Vero cells after treatment of AMPs through a cell viability MTS assay. Vero cells were exposed to each of the nine peptides with doses ranging from 0 to 50 µM for 48 h. Among the AMPs, five showed cell viability of more than 50% compared with untreated controls (peptide concentrations at 0 µM) at the highest dose tested (EC_50_ > 50 µM). These five AMPs include RI-10, DASamP2, GF-17, BMAP-18, and GI-20 (Figure 2A). In contrast, LL-37, GI-20D-form, 17BPIHE2, and merecidin exhibited varying levels of cytotoxic effects with EC_50_ ranging from 4 to 20 µM. To test the anti-ZIKV effects of the AMPs, we chose the doses of 0.4, 2, and 10 µM on the basis of cytotoxicity and early work that showed their effective treatment concentrations (32). ZIKV infection was tested at 48-h post-infection because our earlier work showed that infection causes significant cytotoxicity beyond this time point (27). All AMPs except RI-10 showed dose-dependent decrease of ZIKV RNA compared with untreated controls (Figure 2B). Specifically, Merecidin, LL-37, GI-20, and GI-20 D-form significantly decreased ZIKV RNA at 0.4 µM. DASamP2, GF-17, LL-37, BMAP-18, GI-20, and GI-20 D-form significantly decreased ZIKV RNA at 2 µM. All peptides except RI-10 significantly decreased ZIKV RNA at 10 µM. Taken together, these data demonstrate that eight out of the nine selected AMPs inhibit ZIKV infection in Vero cells.

### Cathelicidin-Derived AMPs Inhibiting ZIKV Infection in Primary Human Fetal Astrocytes

**Figure 3:**
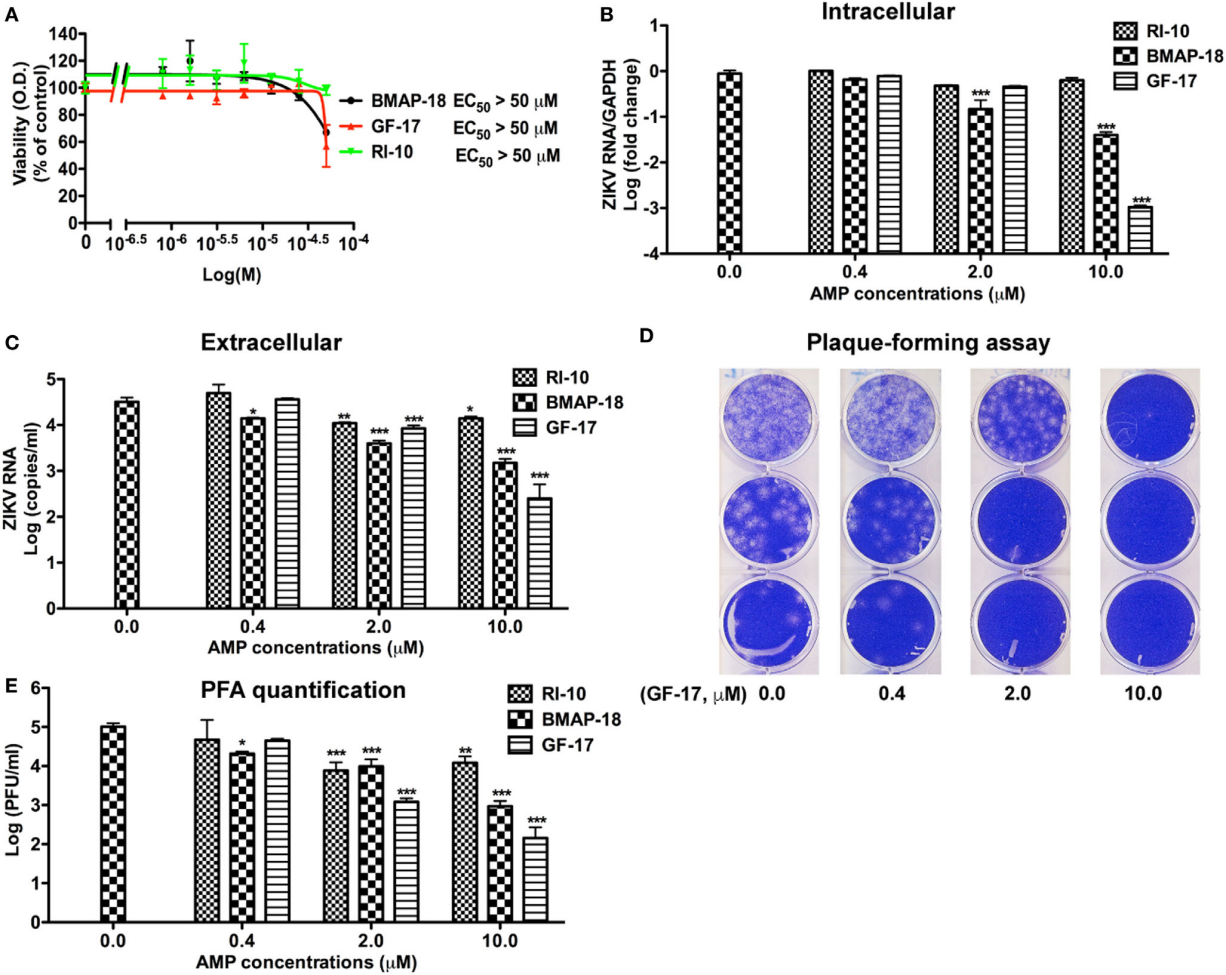
Antimicrobial peptides derived from cathelicidins inhibit Zika virus (ZIKV) infection in human fetal astrocytes. **(A)** Human fetal astrocytes were treated with doses of the selected peptides ranging from 1 to 50 µM. After 24 h, the cultures were washed and treated with same doses of peptides in fresh medium for another 24 h. Cell viability was determined by a colorimetric MTS assay. Results shown are the averages ±SD of experiments performed in triplicate. Data were analyzed by nonlinear regression and assessed the EC50 for each of the antimicrobial peptides. **(B–E)** Astrocytes were treated with doses of antimicrobial peptides ranging from 0.4 to 10 µM for 2 h then infected with ZIKV at the MOI of 0.5. After 24 h, the cultures were washed and treated with same doses of antimicrobial peptides in fresh medium for another 24 h. At the experimental endpoint, intracellular ZIKV RNA **(B)** was determined through real-time RT-PCR in total cellular RNA. Data were normalized to GAPDH and presented as fold change (in log scale) compared with ZIKV group. Extracellular ZIKV RNA [**(C)**, in log scale] was determined through real-time RT-PCR using total RNA isolated from cell-free supernatants. ZIKV virion levels (in log scale) were determined by adding cell-free culture supernatants to Vero cells and overlaid with Agar gel in viral PFA **(D)**. Viral plaques were manually counted and calculated as PFU/mL **(E)**. * *p* < 0.05, ** *p* < 0.01, and *** *p* < 0.001, as compared with the ZIKV group without peptide treatment (ANOVA, *N* = 3).

Since significant cytotoxicity could interfere with the interpretation of the anti-ZIKV activity by AMPs. We treated Vero cells for 2 days (Figure S2A in Supplementary Material) and 4 days (Figure S2B in Supplementary Material) with the 9 AMPs at 10 µM and evaluated the cytotoxicity through the MTS assay. Consistent with Figure 2A, GF-17, RI-10, and BMAP-18 caused minimal cytotoxicity to the Vero cells. Based on these cytotoxicity data, we excluded 17BIPHE2, Merecidin, LL-37, GI-20, and GI-20D-form from further testing. GF-17 and BMAP-18 both belong to the family of cathelicidin. Therefore, we decided to investigate how cathelicidin-derived GF-17 and BMAP-18 impact ZIKV infection. The inactive peptide RI-10 was included as a negative control. Since human fetal astrocytes are a natural host of ZIKV, we tested the effects of GF-17 and BMAP-18 treatment in this cell host. Cytotoxic effects of BMAP-18, GF-17, and RI-10 on human astrocytes were evaluated and no obvious cytotoxicity was observed over 2 days (EC_50_ > 50 µM, Figure 3A; Figure S3A in Supplementary Material) and 4 days (Figure S3B in Supplementary Material) of exposure to them. Therefore, consistent with the data in Vero cells, BMAP-18, GF-17, and RI-10 are not cytotoxic to human fetal astrocytes.

Next, we tested antiviral effects of RI-10, BMAP-18, and GF-17 in ZIKV-infected human fetal astrocytes that we previously characterized (27). Pre-treatments with GF-17 and BMAP-18, but not RI-10, decreased intracellular ZIKV RNA in a dose-dependent manner (Figure 3B). Both GF-17 and BMAP-18 showed most significant inhibitory effect (*p* < 0.001) at the concentration of 10 µM. At 2 µM, GF-17 and BMAP-18 reduced intracellular ZIKV RNA to 46 and 15% of the untreated ZIKV group, respectively. At 10 µM, GF-17 and BMAP-18 reduced intracellular ZIKV RNA to 1 and 4% of the untreated ZIKV group, respectively. The inhibitory effects of GF-17 and BMAP-18 were also demonstrated on extracellular ZIKV RNA in the culture supernatants. For BMAP-18, the level of ZIKV RNA was reduced to 5.6% at 2 µM (*p* < 0.01) and 1.3% at 10 µM (*p* < 0.01). For GF-17, the ZIKV yield was reduced to 16.7% at 2 µM (*p* < 0.05) and 0.1% at 10 µM (*p* < 0.01) (Figure 3C). Consistent with the ZIKV RNA data, ZIKV virions were reduced by GF-17 and BMAP-18, but not RI-10, in a dose-dependent manner (Figures 3D,E). Taken together, these data demonstrate that GF-17 and BMAP-18 inhibit ZIKV infection in primary human fetal astrocytes.

**Figure 4:**
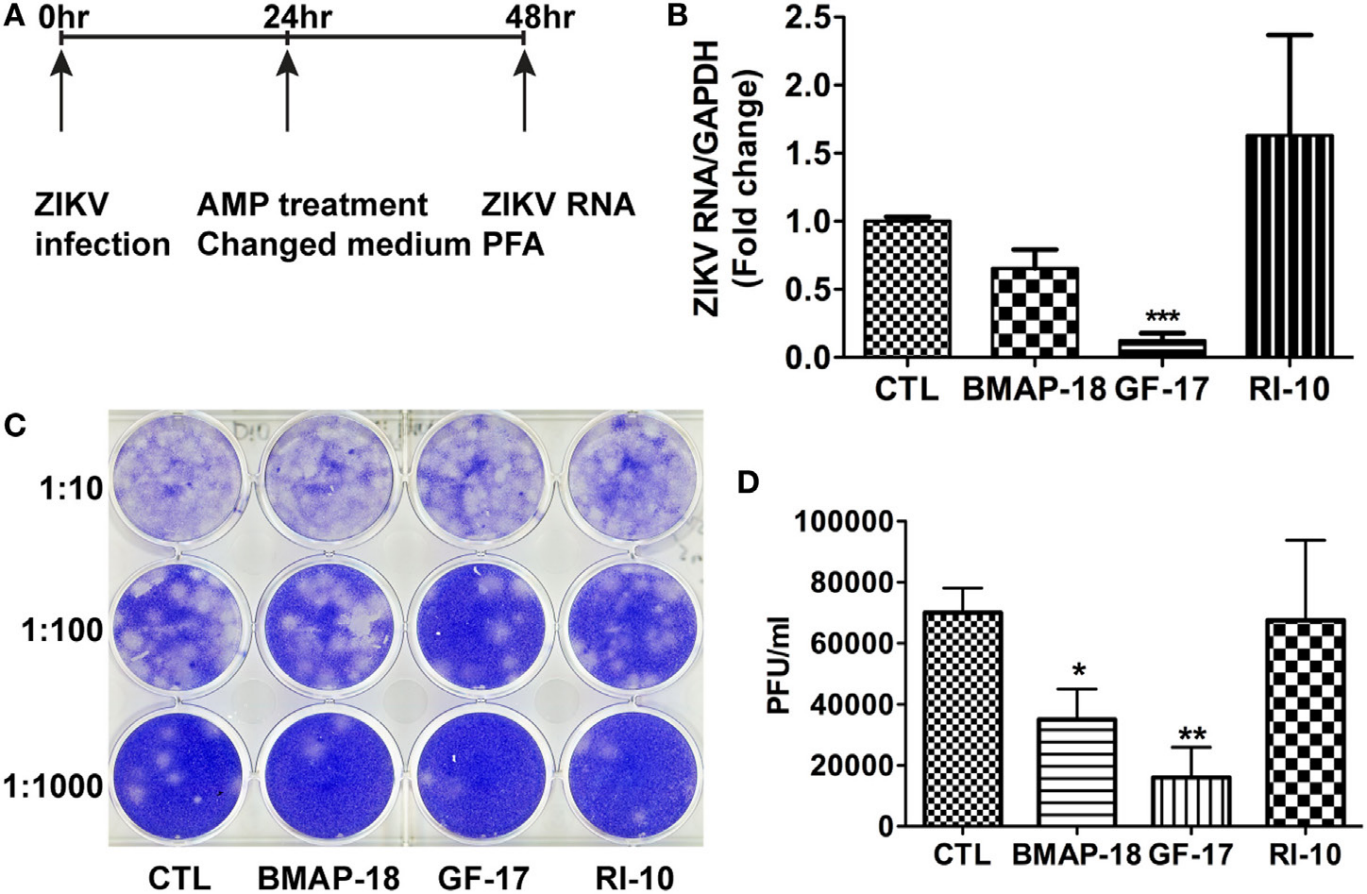
Antimicrobial peptides treatment post-infection is still effective in inhibiting Zika virus (ZIKV) infection. **(A)** Primary human fetal astrocytes were infected with ZIKV at the MOI of 0.5 for 24 h before antimicrobial peptide treatment at 10 µM. **(B–D)** At 24-h post-antimicrobial peptide treatment, RNA was isolated from cells and ZIKV RNA was determined through real-time RT-PCR. Data were normalized to GAPDH and presented as fold change compared with ZIKV group **(B)**. Cell-free supernatants were collected and subjected to PFA for determination of viral titer **(C,D)**. CTL, control. * *p* < 0.05, ** *p* < 0.01, and *** *p* < 0.001 as compared with the ZIKV group without peptide treatment (ANOVA, *N* = 4).

To test the therapeutic potential of cathelicidin-derived AMPs against ZIKV, we changed the treatment of AMPs from pre-infection treatment to 24-h post-infection treatment. ZIKV RNA was determined at 24 h after AMP treatment (Figure 4A). Post-ZIKV treatment with GF-17 for 24 h significantly decreased ZIKV RNA compared with the untreated ZIKV group [*p* < 0.001; Figure 4B)]. ZIKV RNA in BMAP-18-treated group also trended downward; however, the difference was not significant (*p =* 0.063). Also, at 24 h after AMP treatment, Zika virions were quantified through plaque-forming assay; fewer virions were found in BMAP-18- and GF-17-treated groups, compared with those of untreated ZIKV group (Figures 4C,D), suggesting that similar to pre-infection treatment, post-infection treatment with BMAP-18 or GF-17 also inhibits ZIKV infection.

### AMPs Inhibiting ZIKV at 2-h Post-Infection

**Figure 5:**
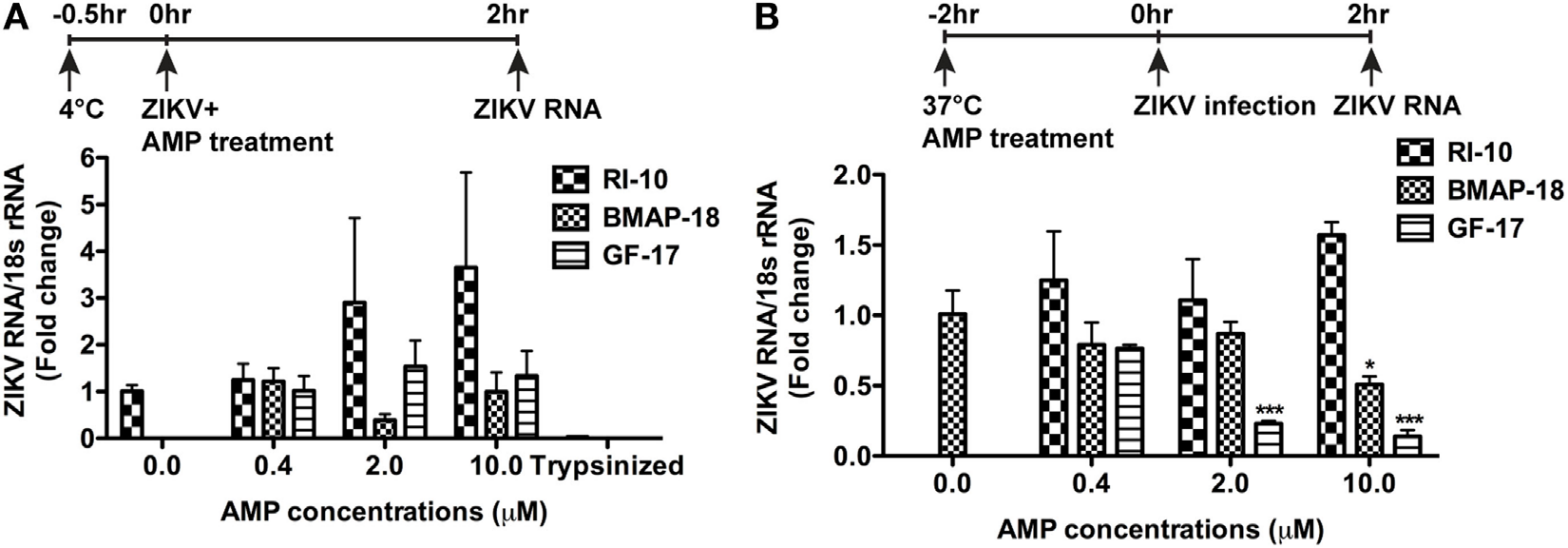
Antimicrobial peptides do not affect Zika virus (ZIKV) viral attachment but inhibit early ZIKV infection. **(A)** Primary human fetal astrocytes were pre-incubated at 4°C for half an hour and then treated with antimicrobial peptides along with ZIKV infection at the MOI of 0.5 for 2 h. Cultures were then washed with fresh medium for three times. ZIKV RNA was determined through real-time RT-PCR in total cellular RNA. Data were normalized to 18-s rRNA and presented as fold change compared with the ZIKV group. The trypsin digestion group readily removes any attached virions and thus serves as positive control. **(B)** Primary human fetal astrocytes treated with antimicrobial peptides for 2 h at 37°C then infected with ZIKV at the MOI of 0.5. After infection for 2 h, cultures were washed with fresh medium for three times and RNA was isolated from whole cells. ZIKV RNA was determined through real-time RT-PCR. Data were normalized to 18-s rRNA and presented as fold change compared with ZIKV group. * *p* < 0.05 and *** *p* < 0.001, as compared with the ZIKV group without peptide treatment (ANOVA, *N* = 3).

To determine the mechanism(s) of AMP-mediated ZIKV inhibition, we first investigated the ZIKV attachment and viral entry. For viral attachment assay, astrocytes were cultured at 4°C for half an hour before the addition of ZIKV and peptides, and the total RNA was harvested after 2 h at 4°C. An extra group of astrocytes was trypsinized to remove any attached virions. This group serves as a positive control for the assay. BMAP-18, GF-17, and RI-10 treatment did not alter the ZIKV RNA levels in astrocytes at 4°C (Figure 5A), suggesting that these AMPs do not affect ZIKV attachment. To determine whether AMPs affect ZIKV entry to the astrocytes, we treated astrocytes with AMPs for 2 h at 37°C before ZIKV infection and collected total RNA at 2-h post-infection (Figure 5B). GF-17 and BMAP-18, but not RI-10, significantly decreased ZIKV RNA (*p* < 0.01, Figure 5B) compared with those of untreated ZIKV group. Taken together, these data indicate that, although GF-17 and BMAP-18 do not affect viral attachment, they impact ZIKV entry at 2-h post-infection.

### Interferon Signaling Is Associated With ZIKV Infection and AMP Effects

**Figure 6:**
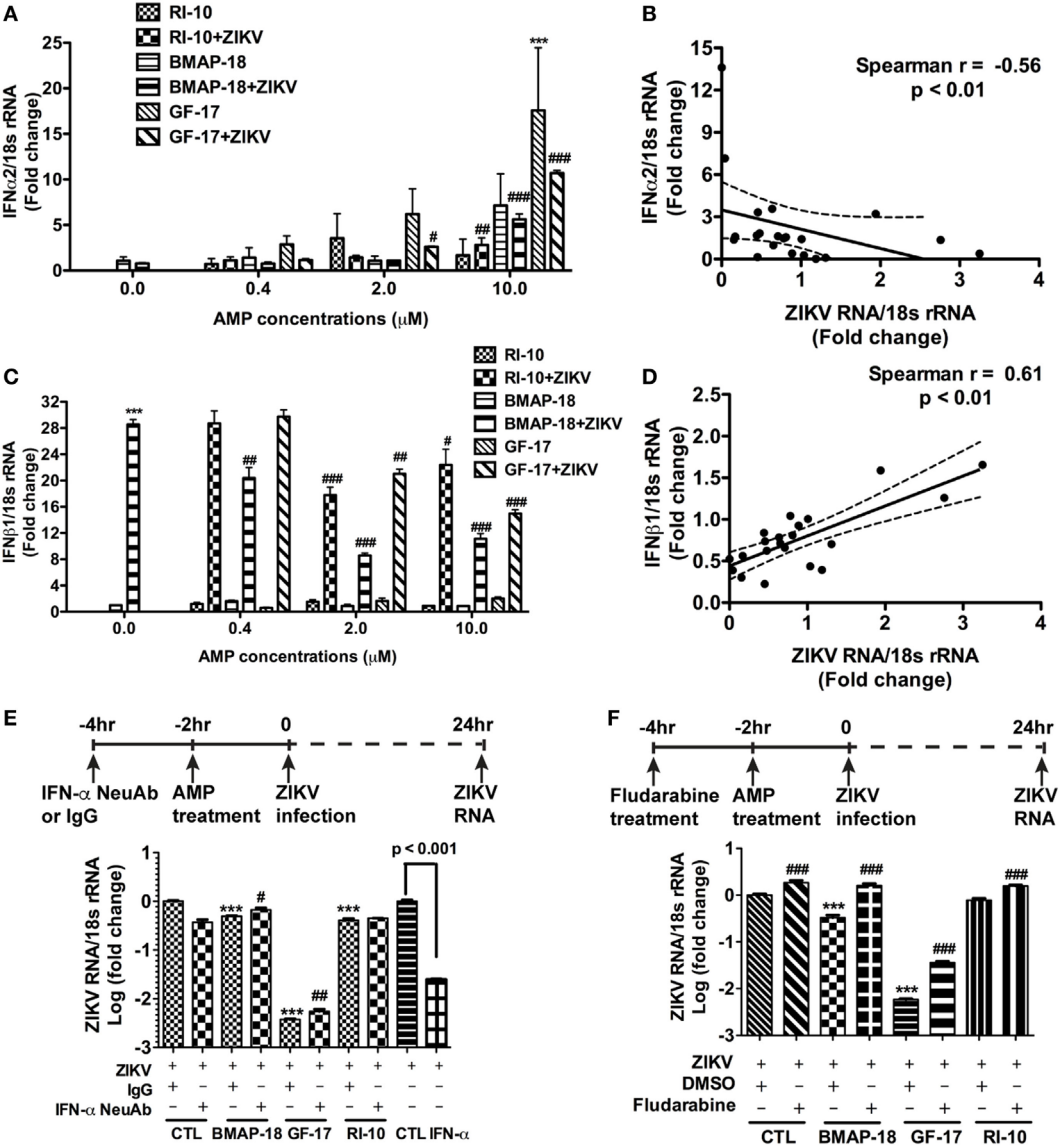
Antimicrobial peptides (AMPs) are negatively correlated with IFN-α2 expression in ZIKV-infected astrocytes. **(A–D)** Astrocytes were infected with Zika virus (ZIKV) at the MOI of 0.5 and treated with doses of AMPs ranging from 0.4 to 10 µM. After 24 h, the cultures were washed and treated with same doses of AMPs in fresh medium for another 24 h. RNA was isolated from cells and the expression of IFN-α2 **(A)** and IFN-β1 **(C)** was determined through real-time RT-PCR. Data were normalized to 18-s rRNA and presented as fold change compared with ZIKV group. Correlation of the IFN-α2 **(B)** and IFN-β1 **(D)** with ZIKV infection levels was determined by Spearman correlation. *** *p* < 0.001, as compared with the uninfected control group. ^#^ *p* < 0.05, ^##^ *p* < 0.01, ^###^ *p* < 0.001, as compared with the ZIKV group (ANOVA, *N* = 3). **(E,F)** Human astrocytes were pretreated with anti-IFN-α neutralizing antibodies **(E)** or 1-µM fludarabine **(F)** for 2 h, then treated with AMPs for 2 h before ZIKV infection at the MOI of 0.5. Rabbit IgG and DMSO were used as control for anti-IFN-α neutralizing antibodies and fludarabine, respectively. At 24 h after infection, total RNA was collected and ZIKA RNA was detected through real-time RT-PCR. Recombinant IFN-α2 was tested for ZIKV inhibition at 1000 units/mL. Data were normalized to 18-s rRNA and presented as fold change in log scale compared with ZIKV group. CTL, control. *** *p* < 0.001, compared with the corresponding ZIKV group treated with control IgG or DMSO; ^#^ *p* < 0.05, ^###^ *p* < 0.001, compared with the ZIKV group treated with the same AMP (ANOVA, *N* = 3).

To further explore the mechanism of AMP-mediated ZIKV inhibition, the levels of IFN-α2 and IFN-β1 during individual AMP treatment with or without ZIKV infection, were determined through real-time RT-PCR (Figures 6A–D). In uninfected fetal astrocytes, control peptide RI-10 did not affect the expression levels of IFN-α2 (Figure 6A). In contrast, GF-17 treatment significantly increased IFN-α2 expression (*p* < 0.0001). Similarly, BMAP-18 also increased IFN-α2 by 7 folds though the difference was not statistically significant (Figure 6A). In ZIKV-infected fetal astrocytes, both GF-17 and BMAP-18, but not RI-10, significantly increased IFN-α2 expression levels in a dose-dependent manner (Figure 6A). Furthermore, the level of IFN-α2 is negatively correlated with ZIKV RNA level in infected cells (Figure 6B). Together, these data suggest that AMPs induce the IFN-α2 expression in fetal astrocytes and the AMP-induction of IFN-α2 likely has a negative impact on ZIKV infection in the cultures. The analysis of IFN-β1 reveals that in uninfected fetal astrocytes, AMP treatment did not affect IFN-1 expression levels. Surprisingly, in ZIKV-infected fetal astrocytes, AMP treatment significantly decreased IFN-β1 expression levels (Figure 6C) and the levels of IFN-β1 were positively correlated with ZIKV RNA level (*p* < 0.01) (Figure 6D). Therefore, these data suggest that IFN-β1 is likely more of a reflection on the ZIKV infection levels and does not play a role in AMP-mediated ZIKV inhibition.

To determine the role of IFNs during AMP-mediated ZIKV inhibition, we first treated astrocytes with recombinant human IFN-α before ZIKV infection. As expected, IFN-α treatment significantly inhibited ZIKV infection (Figure 6E). Next, we used a neutralizing antibody for IFN-α and a small molecule fludarabine known to be an inhibitor for IFN signaling (37). The neutralizing antibody for IFN-α neutralizes multiple subtypes of human IFN-α, including IFN-α2, IFN-α8, and IFN-α21. Inhibition of type-I IFN signaling through neutralizing IFN-α antibody (Figure 6E) or fludarabine (Figure 6F) dampened but did not completely block the GF-17- and BMAP-18-mediated ZIKV inhibition, suggesting that GF-17- and BMAP-18-induced IFN signaling is limiting ZIKV infection in the fetal astrocytes. The modest effect of neutralizing IFN-α antibody also indicates that type-I IFN-independent ZIKV inhibition pathway exists.

### GF-17 Directly Inactivating Zika Viral Particles

**Figure 7:**
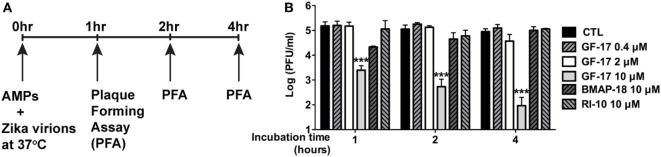
GF-17 directly inactivates Zika virions. **(A)** Experimental scheme. Briefly, Zika virus (ZIKV) virions were directly treated with antimicrobial peptides at 37°C. Part of the inoculum was removed at the indicated time points and serial diluted for the plaque-forming assay to determine the number of infectious Zika virions. **(B)** Quantification of the viral plaques was performed and plaque-forming unit at each of the treatment groups was shown. ANOVA and Bonferroni post-test were performed on all groups. *** *p* < 0.001, as compared with the ZIKV control group (CTL, control).

To determine whether GF-17 and BMAP-18 have a direct effect on ZIKV viral particles, plaque assay was performed on ZIKV incubated with AMPs. GF-17, BMAP-18, and RI-10 (10 µM) was individually mixed with ZIKV and incubated at 37°C for 1–4 h before plaque assay was performed (Figure 7A). These treatment time points were chosen based on a prior report on direct inactivation of vaccinia virions by LL-37 after 2-h incubation (38). After quantification of ZIKV plaques, we found that the effects of GF-17 to inhibit infectious ZIKV virions in the inoculum were both dose- and time-dependent. GF-17 inactivated Zika virions by >95% after 1-h incubation and the effect increased to >99% after 2-h incubation and 99% after 4-h incubation (Figure 7B). Direct incubation of GF-17 and ZIKV at 10 µM and 4 h caused the most virions reduction compared with those of lower doses and shorter time points. In contrast, direct incubation of BMAP-18 or RI-10 with ZIKV did not significantly change the number of infectious virions at all the time points (1, 2, 4 h). Therefore, these data demonstrate that the antiviral properties of GF-17 against ZIKV can be achieved *via* a direct interaction with and inactivation of ZIKV particles.

## Discussion

The ZIKV epidemic in South America in 2015 and 2016 is known to cause severe congenital central nervous system (CNS) malformations such as microcephaly, cerebral atrophy, ventriculomegaly, ocular anomalies, and visual/auditory impairments (39). ZIKV infection is also linked to adult GBS and incidence of laboratory confirmation of a ZIKV infection continues to increase among GBS cases (11, 40, 41). Although several therapeutic approaches have been suggested to be effective against ZIKV, including FDA-approved drugs, type-I IFNs, and live-attenuated vaccine (42–46), currently no vaccination or treatment is available for human use, especially for pregnant women and fetuses (47). In the present study, we demonstrated for the first time that AMPs derived from human and bovine cathelicidins have potent antiviral activity against ZIKV. Humans have only one cathelicidin, which is naturally cleaved to form LL-37. We have further identified that GF-17, which corresponds to the major antimicrobial region (residues 17–32) of LL-37 (28, 48), remains potent against ZIKV and has less cytotoxicity compared with LL-37. Inhibition of IFN signaling partially reversed GF-17-mediated viral inhibition. More importantly, direct incubation with GF-17 specifically reduced the number of infectious Zika virions. Therefore, cathelicidin-derived GF-17 potently inhibits ZIKV through direct inactivation as well as *via* the IFN pathway.

Antimicrobial peptides are able to stimulate antiviral effects through multiple mechanism of action. For example, AMPs with similar secondary structures may have diverging effects on viral replication (49–51). Furthermore, the same AMPs may exert antiviral activity on different viruses through distinct mechanisms (52, 53). Human AMP LL-37 was previously shown to have antiviral activity against HIV-1, influenza A virus, Respiratory Syncytial Virus, and Vaccinia Virus (38, 54–57). However, LL-37 is limited for therapeutic use because of its length of 37 residues, making it costly to chemically synthesize. In addition, pathogens have evolved to compromise the efficacy of LL-37. For instance, pandemic influenza A virus H1N1 2009 (Cal09) is known to resist to LL-37 but not a fragment of LL-37 (54). In the current study, we mainly focused on two cathelicidin-derived peptides: GF-17 and BMAP-18 because of their higher specificity to ZIKV. Our data demonstrate that both GF-17 and BMAP-18 have strong antiviral properties against ZIKV *in vitro* and the antiviral activities can be achieved at low micromolar concentrations. Bovine cathelicidins BMAP-18 was used to show the specificity and uniqueness of human cathelicidins. Our comparison of human and bovine cathelicidins reveals that human cathelicidin might have achieved an evolutional significance to protect humans from viral infection because it works by directly inactivating virus, whereas the bovine cathelicidin-derived peptide is unable to work.

Inhibition of IFN signaling pathway partially blocks the anti-ZIKV effect of BMAP-18 and GF-17. This is consistent with the data that suggest IFN signaling as an important factor regulating ZIKV susceptibility (58, 59). It is unclear how ZIKV continues to replicate despite the apparent induction of type-I IFN by AMPs in the astrocytes. ZIKV may use NS5, a non-structural protein of ZIKV, to antagonize host IFN response by preventing JAK-STAT signaling (60).

One possible mechanism of action by GF-17 is to damage pathogen membrane envelope and interrupt the viral membrane integrity, as previously reported as a carpet-based mechanism (38, 61). Analysis of the sequences of LL-37, GF-17, GI-20, and RI-10 together with their anti-ZIKV activity suggests that residues 1–12 and 33–37 of LL-37 are not essential for the anti-ZIKV effect. In contrast, residues 18 and 29–32 appear to be critical in that antiviral effect are lost if these residues are not present. In addition, residues 13–16 appear to be important in that antiviral effect are modestly weakened if these residues are removed. Analysis of the sequence of 17BIPHE2, Merecidin, and their antiviral activity also suggests that residue R23 is critical since its substitution significantly reduces the antiviral effect. This is consistent with our previous observation that substitution of lysines with arginines increased anti-HIV effects (62). Therefore, these data suggest that residues 17–32 of LL-37 are critical for the optimal anti-ZIKV activities of LL-37.

Few drug candidates are available to safely and effectively reduce viral load and prevent the development of disease after infection (42, 44, 45, 63–72). Compared with the reported drug candidates, AMPs have multiple advantages. First, treatment with GF-17 and BMAP-18 may be a safer choice compared with other drug candidates since the LL-37 is already present in the human body as a component of innate immune system. Second, the antiviral effect of AMPs is rapid, potent, and concentration-dependent. At 10 µM, the reduction of ZIKV RNA by GF-17 and BMAP-18 starts at 2-h post-peptide treatment and ZIKV RNA levels are reduced by over 99% and 95% at 48 h in astrocytes, respectively. Third, the AMPs are effective when applied either before or after ZIKV infection, which indicates the potential use of the peptides as an anti-ZIKV drug both prophylactically and therapeutically. An additional advantage of AMP is that they function through multiple mechanisms. In the event when IFN signaling is compromised by the virus, peptides are still expected to work against the virus *via* a direct inactivation mechanism. However, it is unclear whether GF-17 and BMAP-18 can cross placental and blood–brain barrier. Future studies are needed to evaluate the efficacy of GF-17 and BMAP-18 *in vivo* and how to deliver the peptides to the CNS.

## Conclusion

Cathelicidin-derived AMPs potently inhibit ZIKV infection in Vero cells and human fetal astrocytes. GF-17 mediates anti-ZIKV effect through both a direct inactivation of viral particle and partially through type-I IFN signaling, whereas BMAP-18 inhibits ZIKV through type-I IFN signaling. Strategies based on the peptides documented herein might be useful in halting ZIKV infection both prophylactically and therapeutically.

## Ethics Statement

All experiments for human fetal astrocyte generation were performed with the approval of the Scientific Research Oversight Committee at the University of Nebraska Medical Center (UNMC). Human fetal brain tissues were obtained from elective aborted specimens (gestational age 12 weeks to 16 weeks) following completion of the abortion procedure through collaborative works with the Birth Defects Research laboratory at University of Washington. The protocol is in compliance with all relevant state and federal regulations and is approved by the University of Washington Institutional Review Board (IRB, Protocol no. 96-1826-A07) and UNMC IRB (Protocol no. 123-02-FB). Written informed consent was obtained with all subjects using an IRB-approved consent form at the University of Washington. All consenting subjects were donors of fetal tissue that were 19 years of age or older with clear comprehension. The UNMC investigators do not have access to signed consent forms.

## Author Contributions

MH, YH, GW, and JZ conceived and designed the experiments. MH, HZ, YL, and JZ performed the experiments. MH, HZ, YH, GW, JHZ, and JZ analyzed the data. GW and BT contributed reagents/materials/analysis tools. MH, YH, GW, and JZ wrote the paper. All authors read and approved the final manuscript.

## Conflict of Interest Statement

The authors declare that the research was conducted in the absence of any commercial or financial relationships that could be construed as a potential conflict of interest.
