# Supplementary material for: Cathelicidin-Derived Antimicrobial Peptides Inhibit Zika Virus Through Direct Inactivation and Interferon Pathway
Source: Front Immunol. 2018 Apr 12;9:722. doi: 10.3389/fimmu.2018.00722 (PMC5906549; doi:10.3389/fimmu.2018.00722)
Supplement: Supplementary file 1 [file data_sheet_1.docx]

**Supplementary Figure 1.**


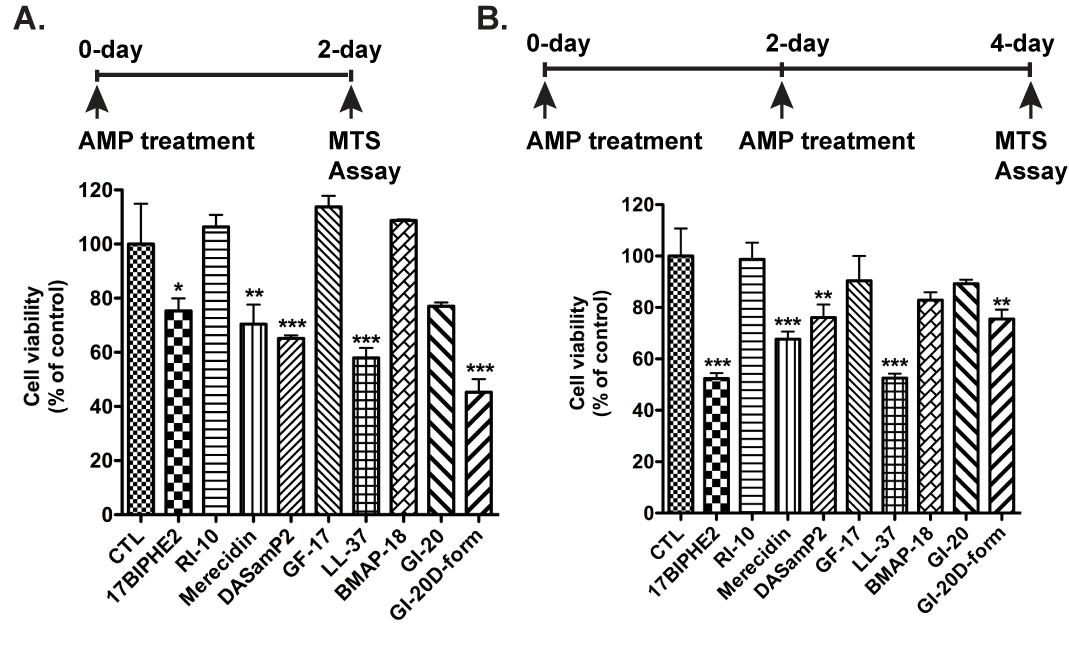


**Figure S1. Vero cell toxicity by AMPs.**

A, B) Vero cells were treated with 10 µM AMPs for 2 days or 4 days as outlined in the figure. For 4-day treatment, Vero cells were repeatedly treated with 10 µM AMPs at 2-day post-treatment. At the experimental end point, cell viability was determined by a colorimetric MTS assay CellTiter 96^®^ AQueous One Solution Assay (Promega, Madison, WI) based on the manufacture’s instruction. Results were normalized as percentage of cell viability in control astrocytes. CTL, control. * denotes p < 0.05, ** denotes p < 0.01, *** denotes p < 0.001 as compared to the control group without peptide treatment (ANOVA, N = 3).

**Supplementary Figure 2.**

**
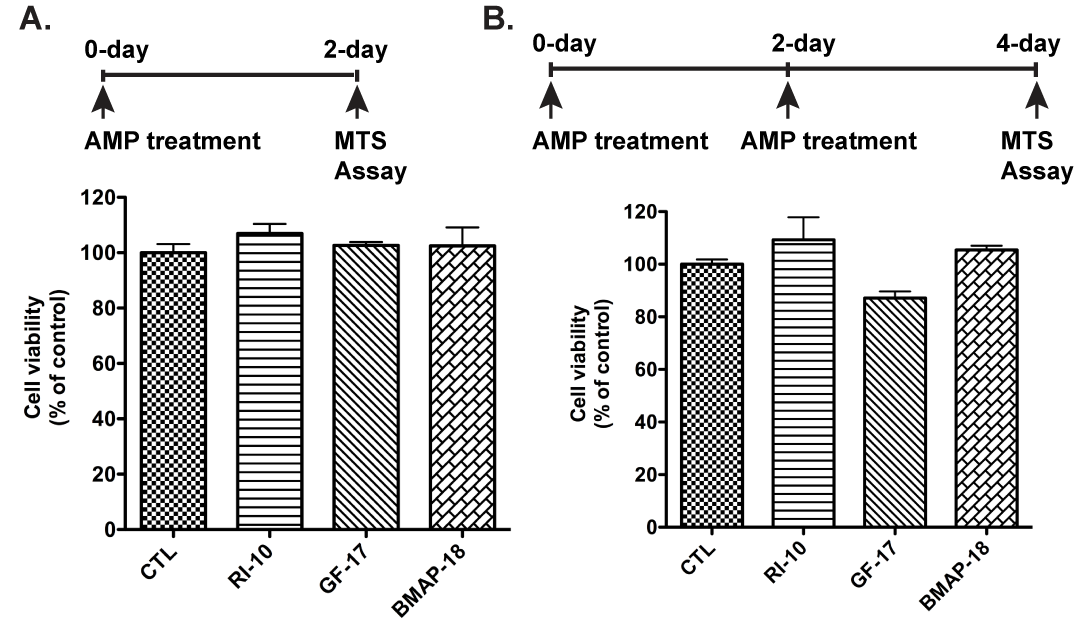
**

**Figure S2. Fetal astrocyte toxicity by the selected AMPs.**

A, B) Human fetal astrocytes were treated with 10 µM AMPs for 2 days or 4 days as outlined in the figure. For 4-day treatment, astrocytes were repeatedly treated with 10 µM AMPs at 2-day post-treatment. At the experimental end point, cell viability was determined by a colorimetric MTS assay CellTiter 96^®^ AQueous One Solution Assay (Promega, Madison, WI) based on the manufacture’s instruction. Results were normalized as percentage of cell viability in control astrocytes. No statistically significant difference was found among all groups for both time points (ANOVA, N = 3).

**Supplementary Figure 3.**

**
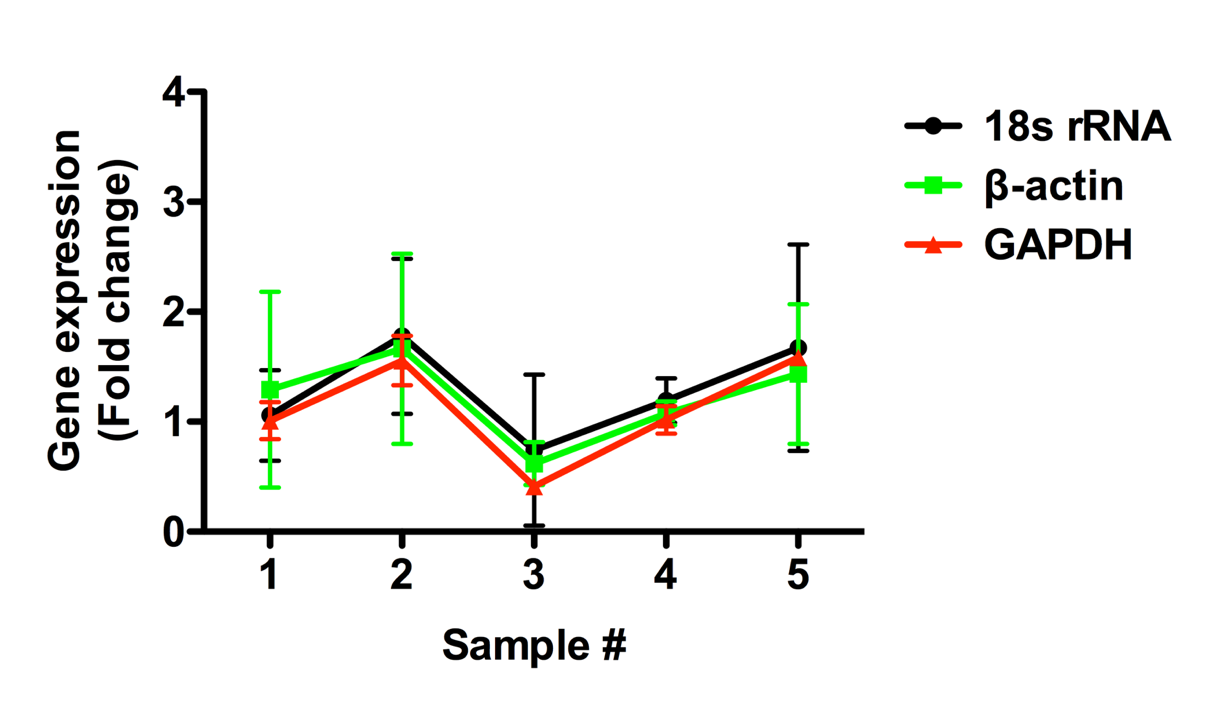
**

**Figure S3. Validation of the reference genes used for real time RT-PCR.**

RNA samples were isolated from five astrocytes treatment groups and the expression of three reference genes, including GAPDH, β-actin, and 18s rRNA, was determined through real-time RT-PCR. Data were not normalized to other reference genes but instead plotted as fold changes to sample #1.
